# A rapid and robust translational model for testicular aging research

**DOI:** 10.1007/s00418-026-02489-3

**Published:** 2026-05-22

**Authors:** N. Schmid, Y. Stepanov, M. Offner, L. Scholz, C. Herrmann, S. Petkov, R. Behr, J. B. Stöckl, T. Fröhlich, A. Mayerhofer

**Affiliations:** 1 Biomedical Center (BMC), Cell Biology, Anatomy III, Faculty of Medicine, Ludwig Maximilian University Munich (LMU), 82152 Planegg, Germany; 2 Laboratory for Functional Genome Analysis LAFUGA, Gene Center, Ludwig Maximilian University of Munich (LMU), 81377 Munich, Germany; 3 Stammzell- und Regenerationsbiologie, German Primate Center, Leibniz Institute for Primate Research, 37077 Göttingen, Germany

**Keywords:** Testis, Peritubular cells, Senescence, Translational model

## Abstract

In humans, testicular peritubular cells (TPCs) form a small compartment surrounding the seminiferous tubules and, as shown previously, undergo age-related changes. How they may contribute to the age-associated decline of testicular functions is not well known. Likewise, the mechanisms of testicular aging in humans are not well examined. This is in part due to the lack of appropriate cellular models. We aimed to establish an aging model of TPCs, which is based on immortalized nonhuman primate (NHP) peritubular cells (iMKTPCs) from *Callithrix jacchus*. As shown previously by a comprehensive proteomic approach, they strongly resemble human TPCs but lack the human cell-intrinsic heterogeneity. Cellular senescence was robustly induced within 10 days upon exposure to 25 µg/mL bleomycin for 24 h. This resulted in increased β-galactosidase activity, enlarged cells, and elevated transcript levels of senescence-associated secretory phenotype (SASP) molecules (*IL1b*, *TNFa*, *CCL2*). The ability to contract upon a stimulus was reduced, as shown in live cell imaging studies. Reduced proliferation among others was indicated by a decreased abundance of proliferating cell nuclear antigen (PCNA), evident in a proteomic analysis, which also revealed massive changes (341 significantly increased and 372 decreased proteins). Bioinformatics analysis indicated a marked reduction in several cell-motility–associated proteins, whereas proteins linked to extracellular exosomes, the cytoskeleton, and lysosomal pathways were increased in abundance. To conclude, bleomycin treatment causes a rapid and robust induction of cellular senescence in a translational testicular cell model of peritubular cells. This model will enable future studies aiming to explore mechanisms and consequences of testicular aging.

**Supplementary Information:**

The online version contains supplementary material available at 10.1007/s00418-026-02489-3.

## Introduction

There is evidence for age-associated changes in the human male gonad (Kaufman et al. 2019; Pohl et al. 2021; Zitzmann 2013; Bier et al. 2025). The alterations may vary but can include marked changes in the peritubular wall regions of the testis. The peritubular wall is a small compartment located between the germ cell compartment in the seminiferous tubules and the interstitial (Leydig cell) compartment. The wall compartment is built by testicular peritubular cells (TPCs) and extracellular matrix (ECM). With age, increases in ECM (fibrosis) can occur, and evidence for reduced cellular contractility of aged TPCs has been provided (Nie et al. 2022). Such changes, which indicate a phenotypic switch of TPCs, are also found in the testes of young men in cases of impaired spermatogenesis (Mayerhofer 2013, 2025). In either case, negative consequences for sperm transport and beyond, possibly on intratesticular paracrine interactions, are likely.

Of note, in old men, an increased body mass index (BMI) may aggravate the reported changes in the peritubular areas (Nie et al. 2022). Thus, confounding issues appear to play roles and, next to obesity, may include diseases, medical treatment, and lifestyle factors, in general. As humans are a long-lived species, as opposed to short-lived rodents, comorbidities and their consequences are difficult to separate from the actual aging processes. This fact particularly renders testicular aging research in humans very challenging, and prospective studies addressing male (reproductive) health over decades are required to better understand age-associated changes and in particular to determine the roles of confounding factors.

Adequate cellular models would likewise be highly desirable. While such models may not allow systemic insights, they may offer ways to examine mechanisms and interactions and might even allow the exploration of potential intervention strategies.

Studies in cultured human TPCs (HTPCs) are possible and have provided new insights into the nature and functions of these cells. The results confirmed the testis-specific smooth muscle character of these cells (Liebich et al. 2022), which contract and relax and thereby transport sperm (Fleck et al. 2021; Liebich et al. 2022). Beyond this well-accepted role, they may contribute to the testicular stem cell niche, for example via glial cell-line-derived neurotrophic factor (GDNF) secretion (Spinnler et al. 2010), and have immunological functions (Mayerhofer 2013, 2025). Due to their location within the testis, they may communicate, for example, via secreted factors with all neighboring cells, including spermatogonial stem cells (SSC), Sertoli cells, Leydig cells, and testicular fibroblasts (Mayerhofer 2013, 2025).

Of note, cultured HTPCs may also serve as a cellular aging model. Repeated passaging over months was reported to result in cellular senescence (Schmid et al. 2019). Cellular senescence, in general, is characterized among others by cessation of proliferation, as well as a specific senescence-associated secretory phenotype (SASP, Hernandez-Segura et al. 2018a, b). In the case of HTPCs, the SASP implied “inflammaging,” which could directly or indirectly influence the SSC. It also resulted in reduced ability of HTPCs to contract (Nie et al. 2022).

Studies in HTPCs, in general, have several drawbacks. As they are patient-derived cells, they are heterogeneous. They furthermore are available only in small numbers, and repeated passaging is very time-consuming, taking months. These points specifically hamper aging studies involving HTPCs.

Evidence for testicular aging has previously also been provided in a nonhuman primate (NHP) species, *Callithrix (C.) jacchus* (Stöckl et al. 2021, 2020). The results indicated that, among other characteristics, their TPCs show age-related changes, including alteration of contractility proteins. Because the access to old *C. jacchus* tissues and especially to senescent testicular cells is very limited, we reasoned that an alternative could be developed, based on immortalized TPCs (iMKTPCs) derived from *C. jacchus*. They are well characterized and, of note, closely resemble human TPCs, as shown by a comprehensive proteomic comparison. In contrast to primary NHP-TPCs or even HTPCs, they are readily available in large numbers and represent a homogeneous cell population (Schmid et al. 2020, 2018).

Different methods have been described which can experimentally induce senescence in cells, in general. They include, next to repeated passaging, radiation, genotoxic drugs, oxidative stress, or demethylating/acetylating agents (Hernandez-Segura, Brandenburg, and Demaria 2018). Among the drugs used is bleomycin, which causes DNA double-strand breaks and thereby induces senescence (Aoshiba et al. 2003; Robles and Adami 1998; Nelson and Kastan 1994). Having tested several of the mentioned options in pilot studies, we used bleomycin and aimed to establish a simple and fast protocol to robustly induce senescence in iMKTPCs. We herein describe the functional and cellular consequences and propose that this translational model may help to advance research on testicular aging.

## Material and methods

### Culture of iMKTPCs

Generation of iMKTPCs and culture conditions have been described previously (Schmid et al. 2020). In brief, iMKTPCs were immortalized by the insertion of human telomerase (hTERT) by PiggyBag transposon (Schmid et al. 2020). The cells were cultured in Dulbecco’s modified Eagle medium (DMEM) high-glucose media (Gibco, Paisly, UK) supplemented with 10% fetal bovine serum (Capricorn Scientific, Ebsdorfergrund, Germany), 1% penicillin/streptomycin (Biochrom, Berlin, Germany), 1× NEAA (Gibco), 1× Glutamax (Gibco), and 0.3 µg/mL of puromycin (Sigma-Aldrich, St. Louis, MO, USA) at 37 °C and 5% (*v*/*v*) CO_2_. For the experiments, iMKTPCs in passages (P) 28–44 were used.

### Induction of senescence with bleomycin

iMKTPCs were cultured in cell culture flasks (T75) until they reached approximately 70–80% confluence. Pilot studies were performed to optimize the concentration of bleomycin sulfate (Cayman Chemical, Ann Arbor, MI, USA). These initial studies included beta-galactosidase staining and cell size measurements. They showed that 25 µg/mL bleomycin sulfate added to the cells for 24 h proved suitable and robustly caused senescence. The respective amount of the solvent dimethyl sulfoxide (DMSO; Invitrogen, Carlsbad, CA, USA) was used as control. After the treatment, cells were washed with phosphate-buffered saline (PBS) and cultured in normal cell culture media for 10 days before analysis by β-galactosidase staining, quantitative polymerase chain reaction (qPCR), proteomics, and functional assays, as described in the following. In addition, cells were cultured for a total of 15 days, after which cell number and size were determined, and qPCR and Western blotting experiments were performed.

### Cell number and cell size measurement

Both the cell size and number of iMKTPCs were measured using the CASY^®^ Cell Counter (Schärfe Systems, Reutlingen, Germany). The cells were trypsinized, centrifuged, resuspended in PBS, and measured as described earlier (Schell et al. 2010).

### Beta-galactosidase staining

iMKTPCs were seeded onto coverslips and staining was performed using a commercial kit (Senescence β-Galactosidase Staining Kit, Cell Signaling Technology #9860, Danvers, MA, USA) according to the manufacturer’s instructions. Evaluation of the staining was done with a light microscope (Zeiss Axiovert; Zeiss GmbH, Oberkochen, Germany).

### Isolation of mRNA, reverse transcription, and qPCR

The total messenger RNA (mRNA) of iMKTPCs was isolated using the RNeasy Plus Micro Kit (Qiagen, Hilden, Germany) following the manufacturer’s instructions. A total of 500 ng mRNA was reverse transcribed using the LunaScript™ RT Super Mix (New England Biolabs, Frankfurt am Main, Germany). A LightCycler^®^ 96 System (Roche, Penzberg, Germany) and Luna^®^ Universal qPCR Master Mix (New England Biolabs), which contains a proprietary double-stranded DNA (dsDNA)-binding intercalating dye-based detection chemistry, were used for the qPCR analysis. Oligonucleotide primers for amplification are listed in Table 1. Quantitative analysis was performed using the 2^−ΔΔCq^ method and normalized to reference genes *YWHAZ* and *PPIA*. Primer pairs, especially those for *IL1B* and *IL6*, were validated in our previous publications (Schmid et al. 2018, 2020) and in the experiments showed comparable quantification cycle (C_q_) shifts between groups. Moreover, single melt peaks were recorded and single amplicon size was verified by gel electrophoresis. The identity of the complementary DNAs (cDNAs) was confirmed upon sequencing. Statistics were obtained using unpaired *t*-tests (two-tailed) of −ΔΔCq values via GraphPad Prism 6.0 software (GraphPad Software Inc.; San Diego, CA, USA); results are shown as means ± SEM. In total *n* = 7 samples were analyzed; non-reverse transcription and non-template reactions served as negative controls.

**Table 1:** Oligonucleotide primer for qPCR studies

Gene name	Nucleotide sequence (5′–3′)		Amplicon size (base pairs)	Accession number
	Forward	Reverse		
*p16*	TAGCCCTACCCGCACAGAT	CGAAGTGTCTCAGAGCCTCA	81	XM_008997757.4
*p21*	TGGAGACTCTGAGGGTCGAAA	GGCGTTTGGAGTGGTAGAAATC	65	NM_001270723.1
*p53*	CCTCAGCATCTTATCCAGGTGG	TGGATGGTGGTACAGTCAGAGC	128	NM_001126118.2
*CCL2*	GCAGCAAGTGTCCCAAAGAA	TGGGGTTATGGAGTGAGTGT	154	XM_002748333.4
*DPP4*	TCACATGGACGGGGAAAGAA	GACCACCACAGAGCAGAGTA	97	NM_001935.4 (TV1)
*IL1b*	GGTTGTCGTGGCTATGGAGA	TTTTGTTGTGCATCCCGGAG	189	XM_002757505.4
*IL6*	AAGAGGTAGCTGCCCCAAAT	AGTGCCTCTTTGCTGCTTTC	145	XM_017975106.1
*TNFa*	ATGAGCACTGAAAGCATGATCC	GAGGGCTGATTAGAGAGAGGTC	217	NM_000594.4
*PPIA*	CTCCTTTGAGCTGTTTGCAG	CACCACAGTCTTGCCATCC	325	NM_006347
*YWHAZ*	CCGCTGGTGATGACAAGAAA	ACACAGAGAAGTTAAGGGCCA	132	XM_054361183.1

### Western blotting

Cells were harvested and used for Western blotting as described previously (Schell et al. 2010). A monoclonal antibody recognizing lamin B1 (1:500; Proteintech, Planegg-Martinsried, Germany; 66095-1-Ig) was used, and an anti-beta actin antibody (1:5,000; Sigma-Aldrich) served as a loading control.

### Contractility measurements

Live cell monitoring of cellular contractility was performed as described previously (Nie et al. 2022). Cells were seeded onto ibidi imaging plates (35 mm µ-dishes; ibidi, Gräfelfing, Germany) and serum-starved for 24 h. Plates were then placed in the imaging chamber at 37 °C and 5% CO_2_ and equilibrated for 30 min. Subsequently, cells were treated with 30% fetal calf serum (FCS, in medium), and the contractile response was monitored for 30 min by acquiring phase-contrast images at 1-min intervals (Zeiss GmbH, Oberkochen, Germany). Cell contractility was quantified by manual measurement of a defined cell area using Fiji (Schindelin et al. 2012). In total, *n* = 20 cells were analyzed. Statistical analysis was performed using a paired *t*-test.

### Sample preparation for proteomics analysis

For proteomics, treated (25 µg/mL bleomycin) iMKTPCs were compared against controls (DMSO) (both *n* = 4). Cells in each sample were lysed in a buffer consisting of 8 mol/L urea (Carl Roth, Karlsruhe, Germany) in 50 mmol/L ammonium bicarbonate (Riedel-de Haën, Seelze, Germany) and sonicated in a Bandelin Sonoplus HD 3200 cup resonator (Bandelin, Berlin, Germany). Lysates were centrifuged through QIAshredder devices (QIAGEN, Hilden, Germany). Protein concentration was determined using the Pierce 660 nm assay reagent (Thermo Scientific, Waltham, MA, USA). Cysteine residues were reduced using 1,4-dithiothreitol for 30 min at 56 °C at a concentration of 5 mM and subjected to carbamidomethylation using iodoacetamide at a concentration of 15 mM (30 min in darkness, room temperature). The digestion of proteins was performed in two steps: (i) Lys C (Fujifilm Wako, Neuss, Germany) in a 1:100 enzyme/protein ratio for 3 h 40 min at 37 °C; (ii) dilution of the samples to 1 mol/L urea and overnight digestion with modified porcine trypsin (Promega, Madison, WI, USA) in a 1:50 enzyme/protein ratio at 37 °C. Dried samples were desalted using ZipTips (Merck KGaA, Darmstadt, Germany) with the protocol recommended by the manufacturer.

### Nano-LC–MS/MS analysis

Nano-liquid chromatography–tandem mass spectrometry (LC–MS/MS) analysis was carried out on an UltiMate 3000 RSLC coupled with a Q Exactive HF-X (both: Thermo Scientific, Waltham, MA, USA). Samples were injected onto a trap column (Acclaim Pepmap™ 100 µm × 2 cm, C18, 5 µm, 100 Å, Thermo Scientific) at a flow rate of 5 μL/min. The LC separation of peptides was performed on an EASY-spray column (Pepmap™ RSLC C18, 2 µm, 100 Å, 75 µm × 50 cm, Thermo Scientific, Waltham, MA, USA) with the flow rate set to 250 nL/min, as follows: a two-step gradient from 3% B (0.1% [*v*/*v*] formic acid in acetonitrile) to 25% B in 160 min followed by a ramp to 40% B for 10 min. Peptides were analyzed in data-dependent acquisition (DDA) mode with the top 15 MS/MS scans selected per cycle.

### Data analysis and bioinformatics processing

Acquired MS spectra were processed using MaxQuant (1.6.11.0) and the *C. jacchus* subset of the UniProt database. Statistical evaluation and visualization were carried out with Perseus v1.6.7.0 (Tyanova et al. 2016) and R (R Core Team 2025). For protein annotation and overrepresentation analysis, the STRING database (https://string-db.org/) was used (Szklarczyk et al. 2023). The mass spectrometry data were deposited to the ProteomeXchange Consortium (www.proteomexchange.org, accessed October 3, 2022) via the Proteomics Identification Database (PRIDE) partner repository with the dataset identifier PXD073445 (Perez-Riverol et al. 2025).

## Results and discussion

**Fig. 1:**
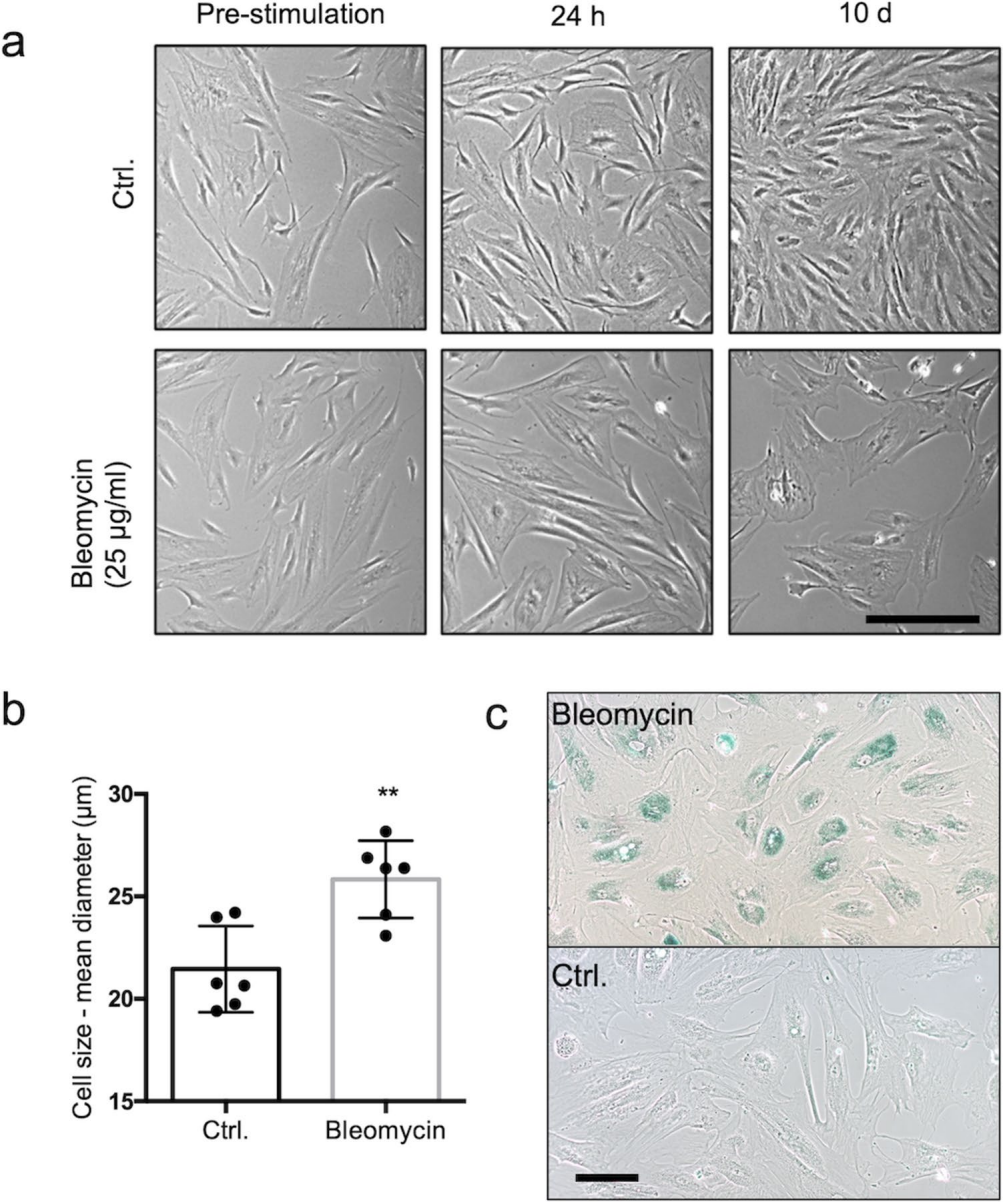
Induction of cellular senescence in iMKTPCs. **a** Light micrographs of different time points (pre-stimulation; 24 h; 10 days) after the treatment of iMKTPCs with bleomycin (25 µg/mL for 24 h) and the respective control cells (Ctrl.). Scale bar: 100 µm. **b** Cell size measurement of bleomycin-treated iMKTPCs 10 days after treatment revealed a significant increase in size (*n* = 6). A paired *t*-test was used for the statistical analysis. Asterisks show statistical significance, *p < 0.05, **p < 0.01; means ± SEM. **c** Light micrographs of senescence associated beta-galactosidase staining 10 days after treatment with bleomycin. The treated iMKTPCs showed blue staining in contrast to the unstained control cells (ctrl). Scale bar: 100 µm

To monitor changes after bleomycin treatment, the cells were observed by light microscopy. Altered flattened cell shapes and significantly increased cell sizes became evident over time (Fig. 1a, b). Beta-galactosidase activity at pH 6, a common senescence marker (López-Otín et al. 2013), became evident 10 days after bleomycin treatment (Fig. 1c). Staining was seen in almost every cell, while control cells lacked staining.

**Fig. 2:**
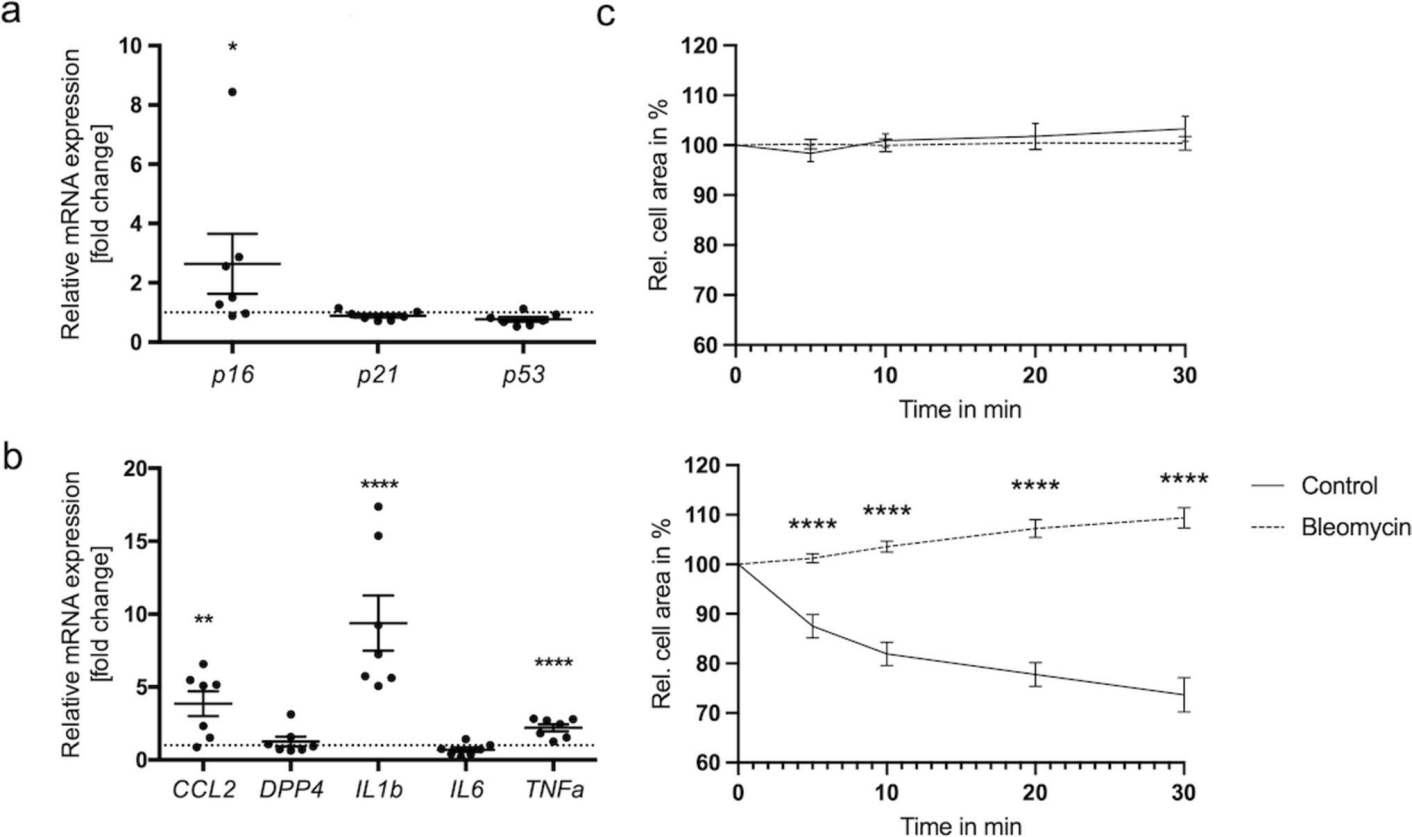
Evaluation of cell cycle arrest marker, SASP markers, and cell contractility. **a** Transcript levels of cell cycle arrest-associated genes *p16*, *p21*, and *p53* were analyzed by qPCR. The level of *p16* was significantly increased. Graphs represent means ± SEM; asterisks show statistical significance, *p < 0.05. **b** SASP marker analysis revealed a significant increase in the transcript levels of *CCL2*, *IL1b*, and *TNFa*, whereas *DPP4* and *IL6* were not significantly changed. Graphs represent means ± SEM, asterisks show statistical significance, **p < 0.01, ***p < 0.001, ****p < 0.0001. **c** Control cells and bleomycin-treated iMKTPCs were observed via live cell imaging, and changes in cellular areas of randomly selected cells were monitored. Cell area was measured after 5, 10, 20, and 30 min and normalized to the cell area at 0 min. Top: Under basal conditions, no changes were observed in cell areas of either group over 30 min. Bottom: After addition of 30% FCS, contraction was triggered in control cells but not in the bleomycin-treated senescent cells. Asterisks indicate statistical significance, ****p < 0.0001. Data are shown as mean ± SEM (%), *n* = 20

At this time point (10 days), the cell cycle arrest markers *p16*, *p21*, and *p53* were evaluated by qPCR. The level of *p16* in senescent iMKTPCs was significantly increased (Fig. 2a). To explore consequences of bleomycin treatment for the cellular SASP and based on previous studies (Schmid et al. 2019), the transcript levels of *CCL2* (C–C motif chemokine ligand 2; MCP-1), *DPP4* (dipeptidyl peptidase 4, denosine deaminase complexing protein 2), *IL1b* (interleukin 1 beta), *IL6* (interleukin 6) and *TNFa* (tumor necrosis factor) were analyzed. The levels of *CCL2*, *IL1b*, and *TNFa* were significantly increased (Fig. 2b). These molecules may contribute to an inflammatory environment within the aging testis and affect the surrounding cells.

As seen in the Online Resource (Supplementary Figures S1 and S2), important senescence signs, namely, larger cell size and increased levels of *p16*, *IL1b*, and *CCl2*, also persisted after 15 days.

As shown by Nie et al. (2022), senescence of HTPCs impairs HTPC contractility. Diminished contractility could then, in turn, impair intratesticular sperm transport. Contractility measurements using single cell observation (Fig. 2c) were therefore carried out, as described (Nie et al. 2022). Under basal conditions, cellular contractions were not observed. However, the ability of senescent iMKTPCs to contract in response to a nonspecific stimulus (FCS) was significantly reduced.

**Fig. 3:**
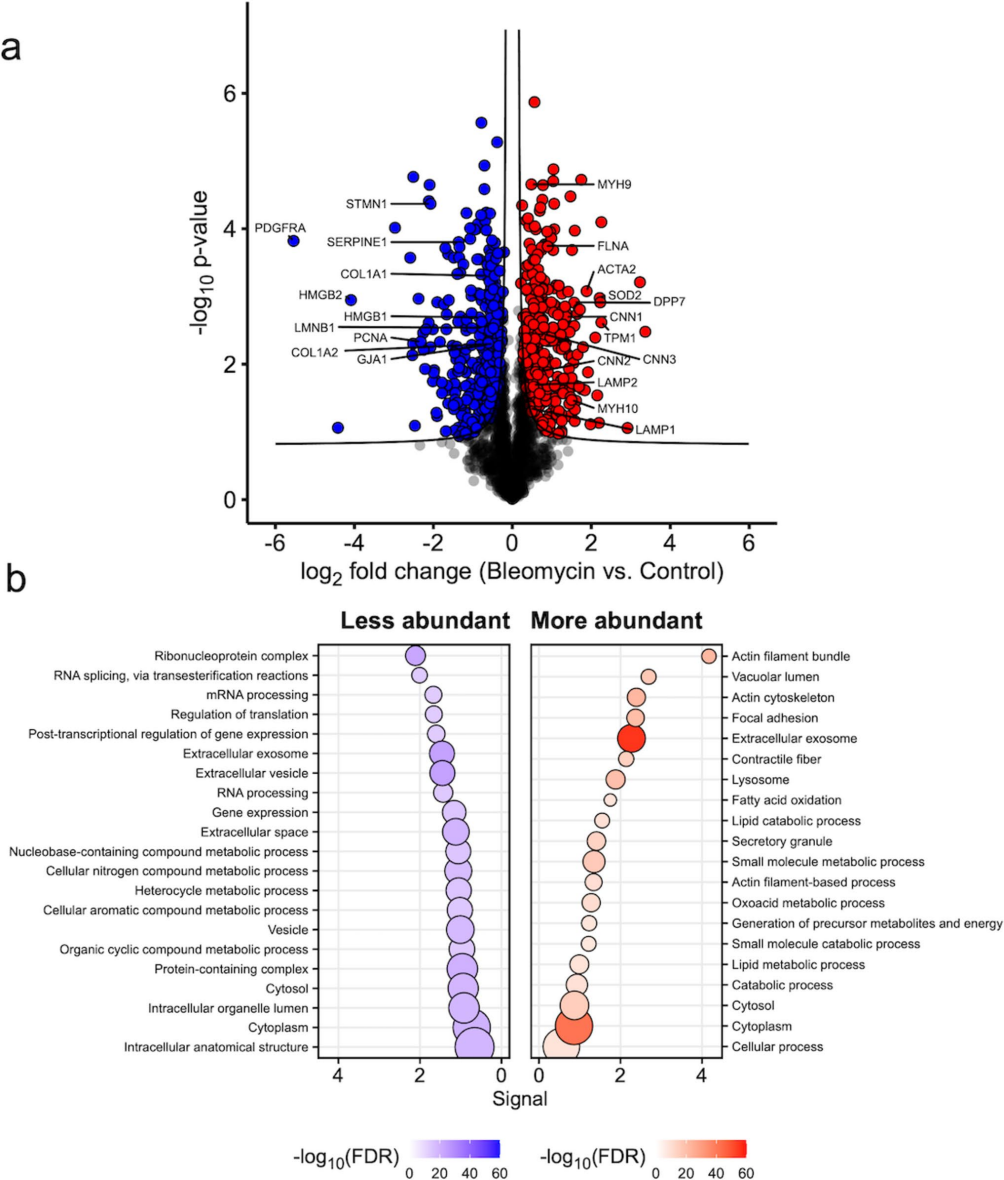
Proteomics analysis of bleomycin-treated iMKTPCs. **a** Volcano plot of bleomycin-treated iMKTPCs compared to control iMKTPCs. Proteins in blue and red represent a decreased or increased abundance in bleomycin-treated iMKTPCs, respectively. Proteins were tested for differential abundance using a modified *t*-test (*S*_0_ = 0.1). A false discovery rate of < 0.05 was enforced using a permutation-based cutoff curve. **b** Overrepresentation analysis using STRING database. More and less abundant proteins were analyzed separately. The 10 most significant terms of both Gene Ontology (GO) biological process and cellular component were selected. FDR = false discovery rate; signal is calculated as weighted harmonic mean between enrichment and −log10(FDR)

A quantitative proteome analysis of the cells (*n* = 4/group) was performed (3439 quantified cellular proteins). The results of LC–MS/MS showed massive changes in protein abundance, with 341 significantly increased and 372 decreased proteins (Fig. 3a, b; Supplementary File S1).

The group of the more abundant proteins includes the actin-binding proteins filamin A (FLNA), several collagens (3A1/8A1), and contractility-related proteins such as CNN1/2/3 (calponins), ACTA2 (smooth muscle actin), TPM1 (tropomyosin 1), and MYH9/10 (myosin heavy chain). Further, DPP7 (dipeptidyl peptidase 7), SOD2 (superoxide dismutase 2), and the lysosomal proteins LAMP1/2 (lysosomal-associated membrane protein) were increased in abundance.

Less abundant proteins after bleomycin treatment included the proliferation marker PCNA (proliferating cell nuclear antigen), resulting in agreement with reduced proliferation. Further, the gap-junction protein GJA1 (connexin 43), the microtubule-regulatory protein STMN1 (stathmin), the collagens COL1A1/2, HMGB1/2 (high mobility group box), SERPINE1 (serpin family E member 1; plasminogen activator inhibitor), and PDGFRA (platelet-derived growth factor receptor alpha) were lower (see Table 2). Of note, LMNB1 (lamin B1) was also significantly lower (Fig. 3a). Reduction of this nuclear envelope marker is an accepted sign of cellular senescence (Freund et al. 2012). The result was confirmed by Western blotting in an additional sample and also persisted for longer times (15 days; Supplementary Figure S2).

**Table 2:** List of top 20 proteins increased or decreased in abundance

Gene names	Protein names	UniProt accession	l2FC	*p*-value
RPL39	Large ribosomal subunit protein eL39	F6S7T1	3.37	3.32E−03
CRYAB	Alpha-crystallin B chain	A0A2R8P261	3.23	6.16E−04
H1F0	Histone H1.0	F7IAQ5	2.92	8.75E−02
TPM1	Tropomyosin 1	F6ZSG9	2.26	2.41E−03
NCEH1	Neutral cholesterol ester hydrolase 1	A0A2R8P216	2.25	7.99E−05
ITGA1	Integrin alpha-1	F7AR09; F7HXE3; F7HXE6	2.23	1.22E−03
SOD2	Superoxide dismutase [Mn], mitochondrial	Q8HXP0; F6PSK1; F6QRF1; F6RMH9; F6PTB4	2.21	1.06E−03
	Matrix-remodeling-associated protein 7 helical domain-containing protein	A0A2R8MTY2; A0A2R8PK69	2.20	7.35E−02
LYPLA1	Acyl-protein thioesterase 1	F6R2X2	2.15	2.89E−02
GNA11	Guanine nucleotide-binding protein subunit alpha	F7I7E6	2.10	4.05E−03
C10orf116	Adipogenesis regulatory factor	F6U3V0	1.98	7.72E−02
C1orf198	Chromosome 1 open reading frame 198	A0A5F4W3M8; F6YTF5; A0A5F4W810	1.92	1.32E−02
ACTA2	Actin alpha 2, smooth muscle	F7IH64; F6PL61	1.88	8.35E−04
SDF2	Bridge-like lipid transfer protein family member 2	F7DM81	1.84	2.43E−02
SMTN	Smoothelin	F7C8G1; A0A2R8M4G3	1.79	5.59E−03
FLOT2	Flotillin	U3FTR0; F7E667; A0A2R8MN25	1.76	2.33E−02
CSRP1	Cysteine and glycine-rich protein 1	F6QGW2	1.75	1.89E−05
TPM4	Tropomyosin 4	U3EY64; A0A5K1UW01	1.71	1.56E−03
CKB	Creatine kinase B-type	F7IQ67	1.69	2.15E−02
	Uncharacterized protein	F6RFH0	1.68	1.53E−03
STMN1	Stathmin	U3DF36; F7EDZ9; A0A5F4WIY6; F7I6I4	−2.07	4.28E−05
SNRPA1	Small nuclear ribonucleoprotein polypeptide A′	F7IJG0; A0A2R8P2N9	−2.07	5.60E−03
CALB2	Calretinin	F7DD39; F7CUW2	−2.10	2.25E−05
EPS8	Epidermal growth factor receptor kinase substrate 8	A0A5F4W281;F7INU5;F7EWV7	−2.11	3.91E−05
APOB	Apolipoprotein B-100	F7HVJ1	−2.12	2.45E−03
TMEM59	Transmembrane protein 59	F7I1M9	−2.19	3.07E−03
RAB3D	Ras-related protein Rab-3	F7I0E3	−2.21	1.17E−02
TBC1D15	TBC1 domain family member 15	U3BIV6; F7IBQ3; F7I9H4	−2.25	6.13E−03
PSMB7	Proteasome subunit beta	F7DRQ0	−2.26	3.46E−03
PCNA	Proliferating cell nuclear antigen	U3CWY2	−2.32	4.65E−03
THBS2	Thrombospondin-2	A0A2R8MRE4; A0A5K1VKE9; F7BBB2	−2.38	1.08E−03
BLVRB	Flavin reductase	U3CGE9; A0A2R8N085; A0A5F4WCE3	−2.47	8.10E−02
DUT	Deoxyuridine 5′-triphosphate nucleotidohydrolase	F7GPR6; A0A2R8PDH0; U3FNL2	−2.51	1.72E−05
KIAA1199	Hyaluronoglucosaminidase	A0A5F4VXV8; U3DDH2	−2.52	4.97E−03
APLP2	Amyloid beta precursor like protein 2	F7HW62; F7I7Z3; F6UJP1; F7I7V8; F7I340	−2.53	7.36E−03
RPL22L1	60S ribosomal protein L22-like 1	F7HZN0; A0A2R8MQC4	−2.59	2.68E−04
HN1	Hematological and neurological expressed 1 protein isoform 1	U3B1Q3; A0A5F4VZ59	−2.97	9.68E−05
HMGB2	High mobility group protein B2	F7HD57	−4.09	1.13E−03
HSPA6	Heat shock 70 kDa protein 6	F6W7H3	−4.42	8.69E−02
PDGFRA	Platelet-derived growth factor receptor alpha	F6VUA7	−5.54	1.51E−04

The Gene Ontology (GO) analysis pinpointed “cytoplasm” and “extracellular exosome” to be massively changed. Exosomes, like SASP molecules, may represent a means of communication of TPCs with other testicular cells. This topic is to our knowledge not well examined, but recently the importance of whole testis-derived extracellular vesicle for spermatogenesis was shown in mice (Zheng et al. 2025). Additional studies are now warranted to address the cargo of exosomes and examine unexplored interactions with other testicular cells either in organotypic incubation or in isolated cells (e.g., by co-culture with fibroblast; Leydig cells).

The proteome analysis of iMKTPCs showed, among others, increased levels of CNN1, which could have impacted the contractile abilities of the cells. Of note, CNN1 was previously reported to be increased in senescent muscle cells (Du et al. 2014). In support, a previous study examined the proteomic changes in whole testes of old *C. jacchus* (Stöckl et al. 2021) and reported an increased abundance of proteins with inhibitory roles in smooth muscle cell contraction, including CNN1. Clearly, the role(s) of CNN1 for the reduced contractile ability, especially in conjunction with other contractility factors or possibly altered adhesion properties in bleomycin-treated iMKTPCs, will require further studies (Liu and Jin 2016).

The previous study of Nie et al. 2022 also showed that the ECM in the peritubular walls of men increased with age. The proteomic results in iMKTPCs mirror such changes, at least in part, with collagens 3A1/8A1 being increased but COL1A1/2 being lowered.

The proteomic analysis further revealed a number of proteins which, to our knowledge, have not previously been associated with the testis or testicular aging. Their roles therefore remain to be determined. For example, CRYAB (crystallin alpha B) and ADIRF (adipogenesis regulatory factor) are strongly increased in the bleomycin group. Others, including APLP2 (amyloid beta precursor like protein 2), are decreased. They are, however, represented in the Human Protein Atlas (HPA) and expressed by human peritubular cells (https://www.proteinatlas.org/; accessed January 28, 2026). Although some of the observed changes in protein abundance may reflect senescence-associated hypertrophy rather than exclusively active regulatory processes, our results taken together underscore the translational relevance of iMKTPCs and suggest that these proteins may constitute promising targets for future research on the human testis and testicular aging.

In summary, different methods have been described for rapid induction of cellular senescence (Aoshiba et al. 2003; Robles and Adami 1998; Nelson and Kastan 1994). In our study in iMKTPCs, we used a short, 24-h treatment with bleomycin and found that it robustly induced senescence. This approach is simple and fast, and robustly resulted in senescent cells within a few days. Although our results indicate that senescence may also be examined at other, longer time points, we have focused on and explored consequences mainly after 10 days. Fast generation, next to homogeneity of the cellular model and availability of the cell line, is of importance for practical reasons. The newly established cell culture model may thus be suitable for examining mechanisms and consequences of testicular aging. The enrichment of the GO term “extracellular exosome” next to the SASP indicates that this model in particular may be well suited to exploring the unknown influence of senescent TPCs on other testicular cells.

## Supplementary Information

Below is the link to the electronic supplementary material.

Supplementary File 1: Proteomic information: Table with all differentially abundant proteins 10 days after treatment of cells with bleomycin. Supplementary file1 (XLSX 248 KB)

Supplementary Figure S1: a. Microscopic images of control cells and bleomycin-treated cells after 15 days. b Corresponding results showing reduced cell counts and increased diameters after 15 in bleomycin-treated cells. Supplementary Figure S2: a Western Blots (original membranes) for lamin B1 and beta-actin (loading control) of control cells and bleomycin-treated cells after 10 and 15 days. Note the decreased lamin B1 levels after 10 and 15 days. b Results of qPCR measurement showing increased transcript expression levels of p16, IL1B and CCl2 in bleomycin-treated cells after 10 and 15 days (normalized to control = 1). Supplementary file2 (PDF 239 KB)

## Data Availability

The mass spectrometry data were deposited to the ProteomeXchange Consortium (www.proteomexchange.org, accessed on 3 October 2022) via the Proteomics Identification Database (PRIDE) partner repository with the dataset identifier PXD073445 (Perez-Riverol et al. 2025).
