# Supplementary material for: A rapid and robust translational model for testicular aging research
Source: Histochem Cell Biol. 2026 May 22;164(1):38. doi: 10.1007/s00418-026-02489-3 (PMC13197275; doi:10.1007/s00418-026-02489-3)
Supplement: Supplementary file 2 — Supplementary Figure S1: a. Microscopic images of control cells and bleomycin-treated cells after 15 days. b Corresponding results showing reduced cell counts and increased diameters after 15 in bleomycin-treated cells. Supplementary Figure S2: a Western Blots (original membranes) for lamin B1 and beta-actin (loading control) of control cells and bleomycin-treated cells after 10 and 15 days. Note the decreased lamin B1 levels after 10 and 15 days. b Results of qPCR measurement showing increased transcript expression levels of p16, IL1B and CCl2 in bleomycin-treated cells after 10 and 15 days (normalized to control = 1). Supplementary file2 (PDF 239 KB) [file 418_2026_2489_MOESM2_ESM.pdf]

Supplementary information

A rapid and robust translational model for testicular aging research

Schmid N.[1], Stepanov Y.[2], Offner M.[1], Scholz L.[1], Herrmann C.[1], Petkov S.[3], Behr R.[3], Stöckl J.B.[2], Fröhlich T.[2], Mayerhofer A.[1]

[1] Biomedical Center (BMC), Cell Biology, Anatomy III, Faculty of Medicine, Ludwig Maximilian University Munich (LMU), 82152 Planegg, Germany; nina.schmid@mail.de (N.S.); Lina.Scholz@bmc.med.lmu.de (L.S.); mayerhofer@bmc.med.lmu.de (A.M.); Carola.Herrmann@bmc.med.lmu.de (C.H.); mo.off@live.com (M.O.)

[2] Laboratory for Functional Genome Analysis LAFUGA, Gene Center, Ludwig Maximilian University of Munich, 81377 München, Germany; Stepanov@genzentrum.lmu.de (Y.S); stoeckl@genzentrum.lmu.de (J.B.S.); frohlich@genzentrum.lmu.de (T.F.)

[3] Stammzell- und Regenerationsbiologie, German Primate Center, Leibniz Institute for Primate Research, 37077 Göttingen, Germany; SPetkov@dpz.eu (S.P.); rbehr@dpz.eu (R.B.)

List of supplementary information files:

- **Supplementary Figure S1**
- **Supplementary Figure S2**
- **Supplementary File 1: Proteomic information - Table (xlsx)**

Supplemental Figure S1

a

Control cells 15 d | Bleomycin-treated cells 15 d

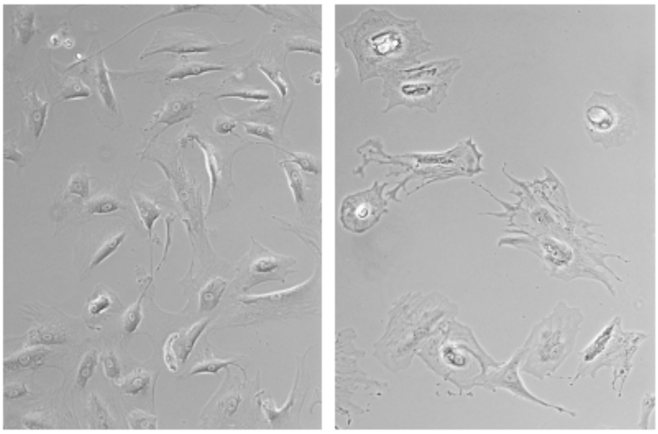

b

Control cells	Bleomycin-treated cells
**Cell count:** 1,02 x 10^6 / well	**Cell count:** 2,96 x 10^5 / well
**Peak cellular diameter:** 24,26 µm	**Peak cellular diameter:** 26,26 µm

Supplemental Figure S2

a

anti-lamin B1 (1:500) | **anti-beta actin (1:1000)**

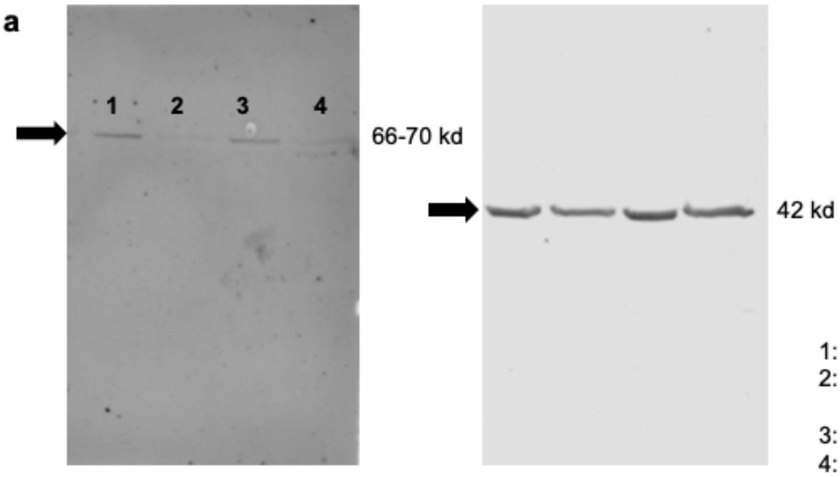

1: d10 Control cells
2: d10 Bleomycin-treated cells

3: d15 Control cells
4: d15 Bleomycin-treated cells

b

Relative transcript expression levels (qPCR)
in bleomyin-treated cells (normalized to controls = 1)

	**d10**	**d15**
p16:	7,46	6,06
*IL1*B:	7,11	5,86
CCL2:	6,45	5,10
